# Cell-Selective Regulation of *CFTR* Gene Expression: Relevance to Gene Editing Therapeutics

**DOI:** 10.3390/genes10030235

**Published:** 2019-03-19

**Authors:** Hannah Swahn, Ann Harris

**Affiliations:** Department of Genetics and Genome Sciences, Case Western Reserve University, Cleveland, OH 44067, USA; hls68@case.edu

**Keywords:** *CFTR*, cis-regulatory elements, enhancers, chromatin architecture, transcription factors, gene editing, CRISPR/Cas9

## Abstract

The cystic fibrosis transmembrane conductance regulator (*CFTR*) gene is an attractive target for gene editing approaches, which may yield novel therapeutic approaches for genetic diseases such as cystic fibrosis (CF). However, for gene editing to be effective, aspects of the three-dimensional (3D) structure and cis-regulatory elements governing the dynamic expression of *CFTR* need to be considered. In this review, we focus on the higher order chromatin organization required for normal *CFTR* locus function, together with the complex mechanisms controlling expression of the gene in different cell types impaired by CF pathology. Across all cells, the *CFTR* locus is organized into an invariant topologically associated domain (TAD) established by the architectural proteins CCCTC-binding factor (CTCF) and cohesin complex. Additional insulator elements within the TAD also recruit these factors. Although the *CFTR* promoter is required for basal levels of expression, cis-regulatory elements (CREs) in intergenic and intronic regions are crucial for cell-specific and temporal coordination of *CFTR* transcription. These CREs are recruited to the promoter through chromatin looping mechanisms and enhance cell-type-specific expression. These features of the *CFTR* locus should be considered when designing gene-editing approaches, since failure to recognize their importance may disrupt gene expression and reduce the efficacy of therapies.

## 1. Introduction

The cystic fibrosis transmembrane conductance regulator (*CFTR*) gene was one of the first genes that was shown to be regulated by elements outside its promoter [1,2,3,4]. At the time, the concept of critical cis-regulatory elements located in introns and intergenic regions was not widely accepted and still challenged dogma that introns contained unimportant DNA sequence. Of course, the Encyclopedia of DNA Elements (ENCODE) project [5,6,7] and many other advances, driven in large part by new technologies based on next generation sequencing protocols, have dramatically altered understanding of genome organization. *CFTR* is a large gene encompassing 189 kb at chromosome 7q31.2 [8]. Although necessary to drive basal levels of gene expression, the *CFTR* promoter is relatively weak and appears to lack tissue-specific control elements. The sequence is CpG-rich, contains no TATA box, has multiple transcription start sites (TSS) and has many binding sites for the transcription factor specificity protein 1 (Sp1) [9,10,11]. Despite this, *CFTR* expression is tightly regulated both during development and within different tissue types [12,13,14,15]. *CFTR* transcript levels are highly variable between different cell types, suggesting that the mechanisms controlling *CFTR* expression may diverge between them.

Cystic fibrosis transmembrane conductance regulator expression was initially thought to be restricted to epithelial cells, specifically epithelial cells within the organs affected by cystic fibrosis (CF) pathology such as the lung, intestine, pancreas, and reproductive tract [13,16,17,18,19]. However, many studies have shown that *CFTR* may also be expressed in non-epithelial cells [20,21]. Additionally, *CFTR* is transcribed in the central, peripheral, and enteric nervous systems [22,23,24,25,26,27,28,29,30,31,32]. Also, Schwann cells were reported to express *CFTR* and CFTR-deficient pigs were suggested to have peripheral nervous system (PNS) deficiencies [30]. Although *CFTR* is expressed in many different cell types, both epithelial and non-epithelial, this review will focus on the regulatory mechanisms controlling expression of the gene in epithelial cells as they are best studied. Here, we discuss both older seminal data and more recent advances that define the chromatin architecture of the *CFTR* locus, reveal multiple cell-type selective cis-regulatory elements within and adjacent to the locus, and identify key activating and repressive transcription factors (TFs). These data have renewed importance at a time when gene editing and replacement are being considered among novel therapeutic approaches for CF. Although *CFTR* is also regulated by post-transcriptional mechanisms including microRNAs, some of which directly target sequences in the 3′ untranslated region (UTR) of the gene, these will not be considered further here as they are reviewed elsewhere [14].

## 2. Common Features of the *CFTR* Locus in All Cell Types

### 2.1. The CFTR Locus Is Organized Within a Topologically Associating Domain

The three-dimensional (3D) chromatin structure has a dynamic and essential role in the regulation of gene expression. On a fine scale, gene regulation occurs at least in part through the physical looping of regulatory elements, such as enhancers to their gene promoters. These looping interactions are thought to be cell-type and locus-specific [33]. On a broader scale, chromatin is organized into topologically associating domains (TADs). TADs are self-associating genomic regions; cis-regulatory elements within one TAD have little to no interaction with genes in neighboring TADs. Therefore, TAD boundaries may represent physical insulators for the genes and regulatory elements contained between them [34,35,36]. These long-range chromatin interactions are measured by many techniques: chromosome conformation capture (3C) [37], circular chromosome conformation capture (4C) and deep sequencing [38], chromosome conformation capture carbon copy (5C) and deep sequencing [39], HiC [40], and chromatin interaction analysis by paired-end tag sequencing (ChIA-PET) [41]. The 3D interactions at the *CFTR* locus were first shown by 3C [42,43,44,45]. Building on these data, 4C-seq demonstrated that the *CFTR* locus is organized into a single TAD with boundaries at −80.1 kb 5′ to the translational start site and +48.9 kb from the translational stop site [46]. These data were confirmed independently by 5C-seq [47,48] and the TAD boundaries were shown to be invariant between cell types [46,47]. Consistent with other TAD boundaries, significant occupancy of the CCCTC-binding factor (CTCF) was observed at the −80.1 kb and +48.9 kb sites [49]. CTCF is an architectural protein involved in chromatin organization that binds to insulator elements and marks TAD boundaries [50,51,52].

### 2.2. The CFTR Locus Contains CTCF-Bound Insulator Elements

In addition to its role in the TAD structure, CTCF may occupy several insulator elements at the *CFTR* locus. These elements, which can block the interactions between an enhancer and a gene promoter, are located at −20.9 kb relative to the translational start site and at +6.8 kb and +15.6 kb to the translational stop site. The sites containing the *CFTR* insulators were initially identified using DNase I hypersensitivity mapping and DNase-seq [42,43,44,53]. CTCF was shown by chromatin immunoprecipitation (ChIP) to occupy the −20.9 kb and +6.8 kb insulators, but not to bind at +15.6 kb [42,53]. The insulator function of +15.6 kb may involve nuclear hormone receptors [53]. The cohesin complex, which occupies a subset of CTCF sites, was also seen to bind at several of the *CFTR* insulator elements, using an antibody specific for the Rad21 component of the complex [42,43,44]. Of note, both the +6.8 kb site and other more distal 3′ elements were cell-type selective [54], consistent with a subset of variant CTCF sites genome wide. Like CTCF, the cohesin complex is involved in chromatin looping and organization. CTCF works in concert with cohesin at ~60%–70% of its sites across the genome [55,56,57]. To determine the significance of CTCF and/or cohesin occupancy in the organization of the *CFTR* locus, small interfering RNA (siRNA)-mediated depletion of both architectural proteins was performed in an intestinal epithelial cell line (Caco2). Loss of CTCF greatly reduced both CTCF and cohesin complex occupancy across the *CFTR* locus, whereas depletion of Rad21 had little impact on CTCF binding. These results suggest the architectural proteins may not always function together, as is observed elsewhere in the genome. Furthermore, CTCF was shown to have a dominant effect on mediating higher order looping of the *CFTR* locus, while cohesin complex was crucial in maintaining stability of the 3D looping at the locus [49]. Interestingly, clustered regularly interspaced short palindromic repeats (CRISPR)/Cas9-mediated deletion of −20.9 enhanced recruitment of CTCF at adjacent sites and thus had little effect on *CFTR* expression [46].

## 3. Cell-Type-Selective CFTR Regulatory Mechanisms

**Figure 1:**
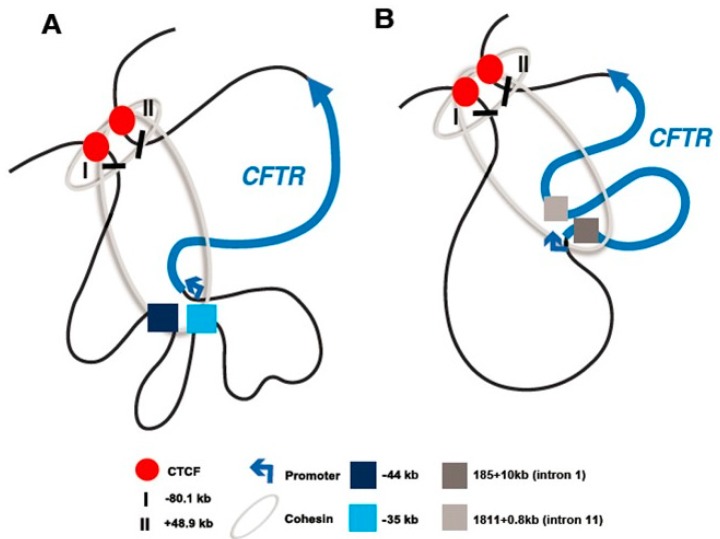
**Schematic of cystic fibrosis transmembrane conductance regulator (*CFTR*) topologically associated domain (TAD) in airway and intestinal cell types.***CFTR* promoter (blue arrow) and gene are shown. TAD boundaries are denoted as “I” (−80.1 kb) and “II” (+48.9 kb). Occupancy of CCCTC-binding factor (CTCF) at the TAD boundaries is shown as red circles. Cohesin complex is shown as gray rings. (**A**) Airway-selective enhancer elements at −44 kb and −35 kb are shown by dark and light blue boxes, respectively. (**B**) Intestine-selective enhancer elements at 185 + 10kb (intron 1) and 1811 + 0.8kb (intron 11) are shown by dark and light gray boxes, respectively. Figures are not drawn to scale.

**Figure 2:**
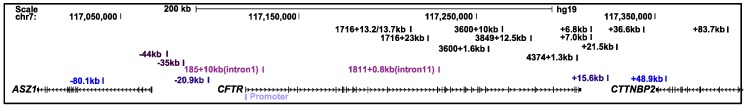
***CFTR* locus and important functional elements.** University of California Santa Cruz (UCSC) genome browser displaying *CFTR* and nearby genes. Airway-selective enhancer elements at −35 kb and −44 kb are shown in dark purple. Intestinal-selective enhancer elements 185 + 10kb (intron 1) and 1811 + 0.8kb (intron 11) are shown in medium purple. Insulator elements at −20.9 kb and + 15.6 kb are shown in dark blue. TAD boundaries at −80.1 kb and + 48.9 kb are shown in medium blue. *CFTR* promoter is shown in light purple. Other key DNase I hypersensitive sites (DHS) are shown in black.

The TAD encompassing the *CFTR* locus is seen in all cells. However, *CFTR* expression is tightly regulated in a cell-type-specific manner due to the recruitment of different cis-regulatory elements (CREs) within and nearby the locus. The interaction between these CREs and the gene promoter generates cell-type-selective 3D conformations of the locus (Figure 1). Here, we discuss some of the known regulatory elements and their activating TFs in different cell types and also consider the significance of the CREs in the context of potential novel therapeutics. Of note, based on our extensive analysis of open chromatin and histone modifications in CF-relevant epithelial cells, there are also other CREs (Figure 2), which are not yet fully understood.

### 3.1. Airway

The major cause of reduced lifespan in CF patients is lung disease, hence the gene-editing protocols that are currently in development are likely to be targeted primarily at this tissue. In this context, understanding the regulation of *CFTR* expression in the airway epithelium, and how this might be disrupted by gene editing protocols, may be critical. Using DNase-chip and subsequently DNase-seq, many airway-selective DNase I hypersensitive sites (DHS) were discovered in lung cell lines that were also seen in primary human airway epithelial cells. These include: DHS at −44 kb, −35 kb, −3.4 kb, in intron 18, 19 and 23, and at +21.5 kb and +36.6 kb (3′ to last exon) [43,44,54]. The DHS at −44 kb and −35 kb were studied in detail to reveal the functions of the CREs they contain. Both have enhancer activity on the *CFTR* promoter in luciferase reporter gene assays and they appear to function cooperatively [43,44,54,58,59], but their enhancer activities are driven by different mechanisms. The core of the −35 kb DHS was mapped using DNase I footprinting and subsequently shown by ChIP to bind the immune mediators interferon regulatory factor 1 and 2 (IRF1/2) and the nuclear factor Y (NF-Y) TF [58]. Additionally, ChIP-seq revealed that this cis-element is enriched for the active histone mark histone 3 lysine 4 monomethylation (H3K4me1) [60]. NF-Y occupancy is required for maintenance of the H3K4me1 modification and so is likely necessary for the enhancer activity of the −35 kb CRE [58]. In contrast, the −44 kb enhancer element was uniquely activated by oxidative stress. It contains an antioxidant response element (ARE), which under normal conditions is occupied by the repressor BTB and CNC homology 1, basic leucine zipper TF (Bach1), and v-Maf avian musculoaponeurotic fibrosarcoma oncogene homolog K (MafK) heterodimers. However, upon exposure to oxidative stress, these repressive factors were displaced by the nuclear factor erythroid 2-like 2 (Nrf2) and *CFTR* expression was activated [59]. This is particularly relevant to CF, as oxidative stress is a hallmark of lung disease pathology [61].

Although there are at least two airway-selective enhancers of *CFTR*, expression in the majority of cells in lung epithelium is significantly lower (~1000–10,000 fold) than that in the intestinal and pancreatic duct epithelium [43,44,62]. This limited transcript abundance in the airway makes designing therapeutics to correct the defective protein quite challenging. A potential explanation for these low levels of *CFTR* transcript in the lung is that repressive TFs are being recruited to the locus in an airway-selective manner. In order to identify transcriptional repressors of *CFTR* in the lung, a siRNA screen was used to deplete ~1500 TFs in a lung adenocarcinoma cell line (Calu-3) [63]. About 50 TFs were, upon depletion, found to elevate *CFTR* transcript levels by at least 2-fold in replicate screens. Among these, knockdown of bromodomain-containing protein 8 (BRD8), ets homologous factor (EHF), krüppel-like factor 5 (KLF5), inhibitor of growth protein 2 (ING2), and nuclear receptor subfamily 2 group F member 2 (NR2F2) had the most robust impact on *CFTR* transcript and CFTR protein levels. Moreover, several of these TFs were also shown to repress *CFTR* in primary human bronchial epithelial (HBE) cells. Of note, both EHF and KLF5 were subsequently shown by ChIP to occupy the −35 kb enhancer element and so may directly repress *CFTR* expression through this site [63]. Understanding these repressive factors is essential, as potential therapeutics could also target their interactions at the *CFTR* CREs.

In contrast to the majority of cells in the airway surface epithelium discussed above, one rare cell type expresses very high levels of *CFTR*. Though they were observed many years ago by mRNA in situ hybridization and immunofluorescence [64,65,66], the likely function of these cells was recently revealed by single-cell RNA sequencing (scRNA-seq). ScRNA-seq of primary human bronchial epithelial (HBE) cells and mouse tracheal epithelial cells documented a high-*CFTR* expressing cell type named “pulmonary ionocytes”. Although these cells only comprise 0.5–1.5% of the airway epithelium, pulmonary ionocytes were shown to be responsible for the majority of *CFTR* activity (~54% in mouse, ~60% in human). Additionally, these cells were shown to co-express the transcription factor forkhead box I1 (*FOXI1*). *FOXI1* expression was suggested to be necessary for *CFTR* expression and loss of FOXI1 was also associated with recognizable CF phenotypes such mucus viscosity and altered fluid composition [67,68]. However, the regulatory mechanisms driving high *CFTR* expression in ionocytes are currently unknown, though in silico predictions (unpublished) suggest they are divergent from other airway epithelial cells. Further experimental data are required to confirm this.

### 3.2. Intestine

Multiple organs in the digestive system are profoundly impaired by loss of CFTR function including the pancreas, bile duct, and intestinal epithelia, among others. As noted above, *CFTR* expression in all these cell types is significantly higher than in the majority of lung epithelial cells. This differential expression is achieved by cell-type selective cis-regulatory elements and their activating TFs. The two best-characterized CREs controlling *CFTR* expression in the intestinal epithelium are intronic: intron 1 (185 + 10 kb) and intron 11 (1811 + 0.8 kb), though other elements both within and outside the gene are also involved.

The 185 + 10 kb intronic CRE is located in a DHS ~10 kb 3′ to end of exon 1 (185 is the last coding base of exon 1) and was found by classical DHS mapping using Southern blots. Its enhancer activity was shown by luciferase assays in the colon carcinoma cell line Caco2 [3]. This CRE was then assayed in the genomic context using a yeast artificial chromosome (YAC) that contained the entire *CFTR* gene [2]. When introduced into Caco2 cells, *CFTR* expression from a YAC lacking the 185 +10 kb DHS (deleted by recombineering), was significantly reduced compared to a control YAC containing the intact *CFTR* gene [2]. To identify the transcription factors governing the enhancer activity of this CRE, DNase I footprinting and electromobility shift assays (EMSA) were performed [69]. These experiments and subsequent ChIP assays showed hepatocyte nuclear factor 1α (HNF1α) binding to the intron 1 185 + 10 kb CRE both in vitro and in vivo [43]. Furthermore, 3C analysis confirmed direct interaction of this enhancer with the *CFTR* promoter [43]. 

Another critical intestinal-specific enhancer of *CFTR* expression is located in intron 11 (legacy nomenclature) at 1811 + 0.8 kb (1811 is the last coding base in exon 11). This enhancer was first identified by DNase-chip within a 1.5 kb DHS in intron 11 [44]. It was also shown to recruit p300, cooperate with other intestinal enhancer elements within *CFTR* and interact with the gene promoter through direct chromosomal looping [44]. Among activating TFs for the intron 11 (1811 + 0.8 kb) CRE are forkhead box protein A1/A2 (FOXA1/A2), hepatocyte nuclear factor 1 homeobox A (HNF1α), and caudal type homeobox 2 (CDX2) [70,71]. These factors were shown to be essential for maintaining high levels of *CFTR* expression in Caco2 cells [70]. 

Although the enhancer elements governing intestinal-selective *CFTR* expression were extensively studied in colon carcinoma cell lines, these may be somewhat influenced by the properties of cancer cells. However, our recent studies (Yin et al., unpublished) suggest there is substantial overlap between the cell line CREs and sites of open chromatin in intestinal organoids, which provide a robust in vitro model of the normal intestinal epithelium [65,72].

### 3.3. Pancreas and Liver

*CFTR* expression is perhaps more abundant in the pancreatic duct epithelium than in any other cell type, consistent with loss of CFTR being associated with profound pancreatic dysfunction. However, very few pancreatic adenocarcinoma cell lines express the *CFTR* gene and so detailed analysis of CREs in this cell type has lagged behind the airway and intestinal epithelium. Earlier work in Capan 1 cells identified DHS in introns 16, 17a, 18, and 20 [4]. In vitro experiments (DNase I footprinting and EMSAs) suggested HNF1, CDX2, and PBX1 bound to the CREs in intron 16 and 17a, however, only PBX1 was shown by ChIP to occupy these sites in Capan 1 cells in vivo [73]. Pancreatic duct cell-specific regulatory mechanisms for *CFTR* are currently being re-examined using functional genomics protocols. Cystic fibrosis liver disease (CFLD) is the third leading cause of mortality in CF patients. In the liver, *CFTR* is expressed at the apical membrane of cholangiocytes within the bile ducts [74] and defective CFTR results in impaired biliary secretion and ductal cholestasis [75]. To date, cholangiocyte-selective CREs for *CFTR* expression have not been investigated.

### 3.4. Male Reproductive Tract

Nearly all (~97%) men with CF are infertile, though not sterile. Loss of CFTR is associated with absence of intact genital ducts, which may be due to early duct obstruction, or a developmental defect impairing duct formation. Congenital bilateral absence of the vas deferens (CVAD) is a common CF-associated diagnosis. The epithelial lining of the epididymis maintains an appropriate luminal environment that is crucial for sperm maturation [76] and CFTR is integral to its function. To determine CREs for *CFTR* in epididymis epithelial cells, we performed DNase-chip on immature human epididymis epithelial cells [44] and subsequently DNase-seq of adult primary human epididymis epithelial (HEE) cells [46] and immortalized, immature epididymis epithelial (REP) cells [77]. These data showed peaks of open chromatin at the *CFTR* locus at a subset of both intestinal and airway CREs, together with novel sites. *CFTR* transcripts are abundant in epididymis epithelial cells [44,78,79] and possibly multiple enhancers are being recruited to drive these high expression levels. Of note, the TFs driving these enhancers may also be different in epididymis cells from the same elements in the intestinal and airway cells. For example, the main form of hepatocyte nuclear factor 1 in intestinal epithelial cells is HNF1α, which enhances *CFTR* expression through multiple cis-elements. In HEE cells, HNF1β is the dominant form and HNF1β ChIP-seq data showed its occupancy at multiple *CFTR* CREs [80]. *CFTR* expression in HEE cells may also be under the control of the androgen receptor [81]. Hormonal control of the *CFTR* locus is not extensively studied to date [82,83].

## 4. Regulation of *CFTR* Expression and its Impact on Gene Editing

Recent advances in the field of gene editing have suggested these protocols as potential treatments for cystic fibrosis, among other diseases. The CRISPR/Cas9 system, which can make targeted double-stranded cuts in DNA, allows for the modification or deletion of any site in the genome [84,85,86]. More recently, the use of base editors is rapidly expanding the field [87,88,89,90,91,92,93]. However, it is not yet known which aspects of the critical 3D structure and interactions at the *CFTR* locus could be disrupted by direct therapeutic modification of the locus. CRISPR/Cas9 protocols have already provided important insights into the functions of CREs and CTCF sites at the locus [46]. The potential to use gene editing to target the *CFTR* gene, CF modifier genes [94,95,96,97,98], or their regulatory elements opens new therapeutic avenues [99]. These may be targeted to accessible sites and specific cell types primarily in the airway and perhaps in the future to other organs affected by CF. Once safety and ethical concerns are overcome, gene editing approaches may also need to account for higher order chromatin structure and the cell-type-selective regulatory networks of *CFTR*, so therapeutic efficacy is not impaired.
